# Nutritional Characteristics, Health-Related Properties, and Food Application of Teff (*Eragrostis tef*): An Overview

**DOI:** 10.3390/ijms26199293

**Published:** 2025-09-23

**Authors:** Boyiza Samson Abebe, Iuliana Aprodu, Daniela Ionela Istrati, Camelia Vizireanu

**Affiliations:** Faculty of Food Science and Engineering, Dunarea de Jos University of Galati, 111 Domneasca Street, 800201 Galati, Romania; samson.abebe@ugal.ro (B.S.A.); iuliana.aprodu@ugal.ro (I.A.); camelia.vizireanu@ugal.ro (C.V.)

**Keywords:** gluten-free cereal, dietary fibers, nutritional compounds, bioactive compounds, health benefits

## Abstract

Teff [*Eragrostis tef* (Zucc.) Trotter] is a globally recognized ancient grain renowned for its attractive nutritional profile and diverse potential applications. Considering its physicochemical characteristics, nutritional value, and probable applications is essential for optimizing its benefits across various food industries. This review aims to comprehensively investigate teff, its physicochemical characteristics, diverse dietary applications, and health benefits. Teff is rich in macro- and micronutrients, making it an excellent addition to various food products. Bioactive compounds, such as flavonoids and phenolic acids, also enhance their functionality. Therefore, teff appears to be a whole grain with favorable technological characteristics and nutritional benefits for various food applications. Also, being gluten-free, teff is acceptable for individuals with celiac disease or gluten sensitivity. Also, it reduces cholesterol levels, supports heart health, stabilizes blood sugar levels, strengthens bone density and strength, and provides immune system support. In conclusion, teff shows excellent potential for developing innovative and nutritious solutions to meet the growing needs of consumers.

## 1. Introduction

Teff (*Eragrostis tef*) is a tropical grain that belongs to the Poaceae family, commonly referred to as the grass family. Of the approximately 350 species in the *Eragrostis* genus, only teff is grown for human use. It is also cultivated in various agroecological regions, including Kenya, Sudan, South Africa, and Nigeria, as well as in several regions across Asia, Europe, and North America [1]. Demand for this crop has recently increased as its grain has become more attractive globally due to its peculiar nutritional benefits. Teff has gained global recognition and is consumed daily by over 30 million people in Ethiopia alone. Its versatility is ideal for food products customized to individuals with dietary restrictions [2].

Teff provides crucial nutrients, including protein, fiber, minerals, vitamins, and phytochemicals, which help sustain stable blood sugar levels. Additionally, teff is free of gluten and harmful antinutrients, making it a healthy alternative for our diet [3,4]. The protein content of teff grains ranges from 12.8% to 20.99% [5]. Teff grains also contain high quantities of fiber (8%) and essential minerals, including iron, calcium, zinc, and magnesium. In addition to their minerals, they also supply vitamins like thiamine, riboflavin, niacin, pyridoxine, and folate, as well as phytochemicals like phenolic compounds and flavonoids [6]. The grains of the crop have phytosterols and saponins but lack any antinutrients. Considering the low glycemic index of 57–74, teff appears to be suitable for individuals diagnosed with diabetes or other digestive issues [7,8,9].

The grains have various culinary uses, including bread, injera, porridge, noodles, cookies, cakes, and beverages. Combining teff flour with other types of flour, such as wheat and sorghum, improves the nutritional quality of the food products [3]. However, the amount of teff added to food products can influence sensory characteristics—food products with lower levels of teff are more favored by consumers [10].

Studying the technological characteristics of teff is crucial because they can influence the quality of food processing, including dough production and bread quality, compared to other regular cereals. The rheological properties and water hydration of teff proteins have been studied, exhibiting potential for various food applications, particularly in baked products [11].

Although further research is required, various health benefits have already been linked with the grain’s typical physical and nutritional properties. For example, some studies have shown that the grain exhibits in vitro antioxidant activity that can increase hemoglobin levels and help prevent malaria, anemia, and diabetes [12,13]. The spectrum of teff’s chemical compounds and functional characteristics has motivated scientists globally to investigate and advance food products that showcase these beneficial properties [14,15]. This review highlights the chemical, nutritional, and functional characteristics of teff while proposing future research directions for its application in the health food sector.

The research was conducted using scientific databases, including ScienceDirect, Scopus, Google Scholar, and Web of Science. Therefore, online databases, books, research reports, and other sources were searched to cover all aspects of knowledge. Sources that lacked updated data or did not serve the article’s purpose were excluded. The obtained data were classified into sections that embrace teff’s chemical and nutritional nature, including proteins, carbohydrates, vitamins, minerals, and biologically active compounds. It also assesses its technological features, applications in the gluten-free market, and potential value-added food products.

## 2. Origin, Diversity, and Distribution of Teff (*Eragrostis tef*)

Ethiopia has diverse agroecological zones characterized by a variety of soil fauna and flora. Due to its favorable environment, the country is a primary center for diversity and a source of economically important crops, including *E. tef* [16]. Teff is an allotetraploid (2n = 4x = 40, AABB), indigenous, self-fertilizing, warm-season grass belonging to the *Eragrostis* genus, Poaceae family [17]. The genus *Eragrostis* is found across Africa, South America, Asia, Australia, Central America, North America, and Europe, with origins of 43%, 18%, 12%, 10%, 9%, 6%, and 2%, respectively [18]. Of Ethiopia’s 54 recognized *Eragrostis* species, 14 are local [18].

**Figure 1:**
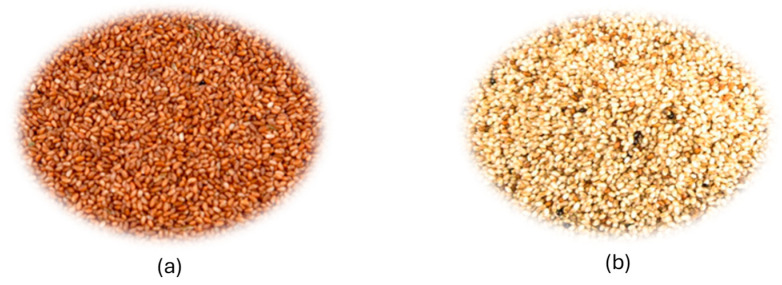
Teff grains (*Eragrostis tef*) of two varieties: (**a**) brown teff and (**b**) white teff.

Ethiopia is a major center of plant origination, supplying many important crops globally, such as *E. tef* and related species [19]. Scientists have confirmed the unique variety of teff in Ethiopia, along with various other native and foreign crops and their wild relatives (Figure 1). This crop’s variation is unique to the world because of its unique origins and domestication [9]. Teff is a native species that comes from a specific place and can only be found in a small area. Teff has been brought to countries like India, but it is still not very important outside of Ethiopia, especially in Eritrea [3,20].

Teff is mostly grown in the Ethiopian highlands, which is a place with a lot of different plants, and it is also grown in Eritrea. People in Ethiopia have been growing teff for thousands of years. Morphological, biochemical, and biosystematic data suggest that *E. pilosa* is the most probable direct wild ancestor of teff. This plant genus is usually found in temperate and tropical areas all over the world, including large parts of Ethiopia [3].

Teff is now grown in many places, including India, Malawi, Zaire, Sri Lanka, Australia, New Zealand, the USA, and South Africa [21,22]. The Ethiopian government has banned the export of teff, which is why it is now being sold outside of Ethiopia. This limit is because food prices are going up and the domestic market is unstable, which makes teff too expensive for most people [3]. This has made people very interested in growing teff outside of Ethiopia, which has led to a big rise in production. Teff production started in the U.S. in 1984, and the amount grown has gone up a lot because more people want this healthy grain. The need comes mostly from Ethiopia and the US, as well as a need for high-quality animal feed [23]. Moreover, teff has recently been introduced to northwestern Europe, specifically in the Netherlands [18].

Understanding the genetic diversity of teff is essential for developing improved varieties with high and stable yields. Extensive research has been carried out to assess the level and patterns of variation within teff germplasm collections, using a range of analytical methods [24,25,26]. There is significant phenotypic and genetic variability in nutritional traits (protein, minerals, phytate, phenolic compounds) and their associated genes, providing valuable opportunities for elucidating the relationships between these traits and identifying allelic variants, useful in developing teff varieties with optimal nutritional profiles [27].

## 3. Nutritional Implications of Teff (*Eragrostis tef*)

This grain contains high amounts of essential nutrients, including iron, calcium, copper, zinc, phosphorus, and potassium, all of which play important roles in establishing a versatile nutritional foundation [28]. This suitable composition, along with its remarkable health benefits, makes teff an attractive option for gluten-free food products for individuals with gluten sensitivity or celiac disease [29].

Further, teff is recognized for its rich content of minerals, protein, lipids, dietary fiber, amino acids, polyphenols, starch, carbohydrates, and vitamins, which are unique to those found in cereal grains [30]. Due to its gluten-free nature and abundance of micronutrients, including calcium, iron, and fiber, teff is a suitable addition to gluten-free food lists [31]. The high fiber content, nutritional properties, and prebiotic properties are important nutritional values of teff [32,33]. Teff is considered a vital grain due to its superior nutrient profile compared to many other common cereals (Table 1).

### 3.1. Carbohydrates

The carbohydrate of teff contains monosaccharides, disaccharides, oligosaccharides, polysaccharides, and fiber. Starch is the major component of teff grain, accounting for 74% to 75.5% of the dry weight [46,47]. This can vary due to genetic factors, soil quality, and weather conditions [8,48]. Starch contains 21–22% amylose, which implies slow digestion and affects its functionality in food [49]. Teff contains a very low-viscosity starch, the primary storage polysaccharide in plants. This starch consists of two types of glucose polymers: amylose and amylopectin. The proportions of amylose to amylopectin influence the physicochemical and functional properties of starch, including gelatinization, elasticity, viscosity, and digestibility. The high gelatinization temperature of teff starch (68–80 °C) can lead to lower susceptibility to enzymatic attack by α-amylase [44]. Amylose content ranges from 20% to 30% of the teff grain, depending on the variety and growing conditions. In addition, most cereal starch have a grain size larger than 0.6 to 1.9 mm, as in teff starch [46]. Specific microorganisms can transform the teff starch during food processing (injera).

Microorganisms can reduce the starch content to 48–58%, depending on the duration of fermentation and the type of microorganisms used [15]. Several factors influence the digestibility of teff starch, including the ratio of amylose to amylopectin, granule size, the degree of gelatinization, retrogradation behavior, and the presence of other components such as proteins, fibers, and other phytochemicals [50]. The digestibility of teff starch ranges from 80.5% to 85.5%, depending on the variety and treatment [51]. Teff starch has a low to medium level of resistant starch, ranging from 1% to 5%, depending on the variety and processing technique. Resistant starches are not digested in the small intestine but are broken down in the large intestine. They act as prebiotics, nourishing beneficial bacteria that inhabit the large intestine. Eating resistant starch can enhance gut health, reduce blood cholesterol levels, and control immune function [7,52].

The functional characteristics of starch are crucial in food systems because they significantly influence product properties, including texture, color, taste, and stability. Teff starch has many benefits, such as being able to absorb water and oil well, swell up, dissolve, emulsify, gel, and paste. These qualities make it possible to make a variety of products, such as alcohol-free wine coolers, fruit ciders, and bar snacks that can be used instead of dairy alternatives [53]. Nonetheless, teff starch can be subjected to hydrolysis or gelatinization via physicochemical and enzymatic techniques, yielding functional attributes such as stability, clarity, viscosity, and resistance to freeze–thaw cycles. Teff starch can also be mixed with other starches or hydrocolloids to make it work better for different uses [3].

### 3.2. Proteins

Proteins are macronutrients that the body needs, and teff has a lot of them, between 12.8% and 20.9%, which is about the same as wheat and corn (Table 1) [54,55]. Glutelins are the main proteins in teff, making up more than 46.6% of its total protein [56]. Teff has a lot of important amino acids, especially lysine, which most grains do not have enough of (Table 2). Studies indicate that teff contains a significantly higher concentration of lysine in comparison to wheat and maize, rendering it a crucial protein source, especially for communities whose diets predominantly consist of cereals [11,38]. Teff does not have the proteins that make gluten, which makes it a great choice for people with celiac disease or gluten intolerance [11].

Teff provides many nutritional benefits. This tiny grain features remarkably varied profiles of amino acids, making it an excellent source of dietary protein. The main component is glutamic acid, which significantly enhances the remarkable protein content of teff [63]. Additionally, the essential amino acid lysine, which is often limited in other cereal proteins, is abundant in teff [64]. Furthermore, other essential amino acids, such as leucine, isoleucine, and valine, are also present in teff’s composition, underscoring its potential to support muscle development and promote overall well-being [56] (Table 2).

### 3.3. Teff Lipids and Their Functional Properties

Most cereals are not a good source of lipids but are utilized in large quantities. The lipids content of this grain is low, and its lipids are mainly unsaturated. Unsaturated lipids are believed to have a positive effect on cholesterol levels, thereby reducing the risk of heart disease. Furthermore, teff lipids contain linoleic and alpha-linolenic acids, which are vital fatty acids essential for synthesizing omega-6 and omega-3 polyunsaturated lipids. From a medicinal perspective, they are crucial for constructing cell membranes and regulating inflammatory processes in humans [43,44].

Various factors influence the fatty acid composition of teff lipids, including the variety, growth conditions, processing methods, and storage conditions. Teff lipids are predominantly composed of unsaturated fatty acids, accounting for 39.91%, and saturated fatty acids, accounting for 20.06% of the total fatty acids [5]. The main unsaturated fatty acids in teff are oleic acid (C18:1), linoleic acid (C18:2), and alpha-linolenic acid (C18:3), which account for 32.4%, 23.8%, and 5–10% of the total fatty acids, respectively [5]. The major saturated fatty acids are palmitic acid (C16:0) and stearic acid (C18:0), which account for 15–20% and 5–10% of the total fatty acids, respectively [43,65]. Table 3 presents information on the fatty acid content of teff, depending on the variety and its role in human health.

Teff grains typically have a lipids content of 2.4–5.1 g/100 g on a dry matter basis, which varies by variety and growing conditions [43] (Table 1). Teff flour contains a slightly lower lipids content, approximately 3–5%, due to the milling process that removes some starch. As a result, the lipid content in teff injera is lower than in other types, ranging from 1.93% to 3.03%, because lipase breaks down lipids during fermentation [66]. The amount of fat in different teff-based products, such as bread, pasta, noodles, sweet and savory biscuits, cakes, muffins, and drinks, depends on the proportion of teff flour used compared to other ingredients [12,43,66]. The fats in teff are important in food because they have special functions. They are stable and do not break down easily over time, which helps the food look good and lasts longer, even when stored for a long time. One special thing about teff fats is that they mix oil and water quickly to make a smooth, even mixture [67]. Also, different methods like physical, chemical, or enzyme-based techniques can change the properties of teff fats, such as how they melt, how they form crystals, and how soft or firm they become [68,69].

### 3.4. Minerals and Vitamin Content of Teff and Their Functional and Nutraceutical Effects

Teff is rich in micronutrients and plays a vital role in various metabolic processes, enzymatic reactions, and physiological functions within the body. This cereal grain has a lot of calcium, phosphorus, magnesium, potassium, iron, and zinc, which are all important for making bones, making blood, using energy, keeping the immune system healthy, and keeping the body’s water balance. Teff also has a lot of thiamine, riboflavin, niacin, vitamin A, and vitamin K, which help the body break down carbohydrates, protect against free radicals, keep skin healthy, improve vision, and help blood clot [7,70].

The micronutrient content of teff grains is affected by the type of teff and how it is grown. There are many things that can change how bioavailable teff micronutrients are. These include different ways to process and cook teff and add other things, like phytates or tannins. Compounds with a higher molecular weight, like those with complex metal ions like iron and zinc, can stop these nutrients from moving from the intestine into the blood. Teff’s phytates, tannins, and oxalates make it harder for the body to take in iron and zinc [71]. Fermentation, soaking, germination, and malting are all ways to process foods that can lower the levels of these compounds while making iron more available to people. Also, when teff is mixed with other foods like meat, dairy, fruits, and vegetables, it can make iron and zinc more available to the body [72]. This may happen by adding more of these nutrients or making them easier to dissolve and absorb [73,74].

The functional properties of teff micronutrients are essential for nutritional applications. Micronutrients affect the color, taste, texture, and shelf life of food. Teff micronutrients may also work as natural antioxidants, stopping the oxidation of lipids, proteins, and carbohydrates [11]. This not only boosts the quality and shelf life of teff products but also enables the micronutrients in teff to function as natural preservatives, hindering the growth of spoilage and hazardous microorganisms [75]. Therefore, teff micronutrients improve both the safety and quality of teff products. They also serve as common supplements, enhancing the dietary value and health benefits of teff products. Moreover, they serve as natural colorants, sharing diverse shades and tones with teff-based products, depending on the type and concentration of micronutrients [11,76].

Table 3 compares the mineral content of diverse teff grain types to that of maize and sorghum from different research reports. Red teff, for example, contains a remarkable amount of iron (7.1–9.3 mg/100 g), which is not found in maize (0.5–2.7 mg/100 g) or sorghum (3.4–4.8 mg/100 g). Therefore, it is an effective solution for iron deficiency [44]. White teff is excellent because it contains a significant amount of calcium, which is found in trace amounts in maize and in some quantities in sorghum. Thus, it is superior to the two cereals; it contains up to 150–180 mg/100 g, which is essential for proper growth [8,44] (Table 4). Additionally, teff is rich in magnesium and phosphorus, which further enhances its nutritional profile. Its significant zinc content differentiates teff from other cereals, stressing its relevance in filling diverse dietary gaps.

Teff is exceptionally abundant in various compounds with nutritional value. It contains various essential vitamins that complement a well-balanced diet. These include vitamin C, niacin, vitamin A, riboflavin, and thiamin [77]. Further, teff is recognized as a source of vitamin B6, vitamin K, vitamin E, and α-tocopherol [78]. These vital vitamins are essential in numerous bodily processes, including supporting the immune system, regulating energy metabolism, and providing antioxidant defenses [11].

Teff’s vitamin content and nutritional characteristics make it a balanced dietary choice that can enhance overall health and well-being. Teff has an exceptional nutritional composition compared to other grains. It is an excellent source of B vitamins, particularly thiamin (Vitamin B1), riboflavin (Vitamin B2), and niacin (Vitamin B3), which are found in high amounts compared to other nutritious grains, such as quinoa and rice [79] (Table 5).

### 3.5. Bioactive Compounds of Teff

Currently, interest in bioactive compounds has risen rapidly, as it is implicit how they can beneficially influence human health. Teff (*E. tef*) could be a potential source of bioactive compounds, including polyphenols, phytosterols, and flavonoids, which contribute to its health benefits [84].

Phenolic acids and flavonoids are found in cereals as free compounds or conjugated with other molecules. Phenols present in whole grains have significant health benefits, helping to reduce the risk of chronic diseases. They also modify the taste and odor characteristics of whole-grain products and serve as an essential indicator of biological activity. Bound phenols may play an important role in the prevention of colon cancer, while soluble phenols, which are rapidly absorbed in the stomach and small intestine, may support overall health [85].

Polyphenols, such as ferulic acid and caffeic acid, are present in high concentrations in teff and possess strong antioxidant properties, which support the reduction in free radicals and lower oxidative stress [44,86]. Flavonoids, such as luteolin and apigenin, increase teff’s antioxidant capacity and also reveal benefits for anti-inflammatory and antimicrobial properties [38]. These compounds are predominantly found in the bran layer, which remains intact even after grinding, making teff an excellent choice for maintaining nutrients compared to refined grains [87].

Studies have shown that the total phenolic content (TPC) in teff grains ranges from 46 to 133 mg GAE/100 g, and the total flavonoid content ranges from 15 to 113 mg QE/100 g [88]. Kotásková et al. highlighted that the content of phenolic compounds in teff is higher in the free fractions (0.9–1.4 mg GAE/g) compared to those in the bound fractions (0.4–0.7 mg GAE/g) [51]. It was also highlighted, in the same study, that the content of flavonoids (0.5 mg RE/g) in the free fractions was greater than in the bound fractions (0.1 mg RE/g) [51]. Other studies reported that the content of total phenolic acids, ranging from 600 to 728 μg/g, was predominantly found in bound fractions. Additionally, the content of phenolic acids in teff is dominated by ferulic acid and p-coumaric acid, both in the free and soluble fractions [84,89]. Also, high levels of flavonoids were detected in both white (1.4–2.1 μg/g) and brown teff (1.7–1.8 μg/g) [87]. Brown teff contains mainly trans-p-coumaric acid, protocatechuic acid, ferulic acid, and gallic acid in the free form and in the bound fraction, quercetin, and catechin. In contrast, white teff is rich in rutin, protocatechuic acid, and ferulic acid, in the free form, and rutin and catechin in the bound form [51,89]. Homem et al. reported similar levels of zeaxanthin, cryptoxanthin, and α-carotene in breads prepared from teff flour as those from wheat flour, thus indicating the presence of these carotenoids in teff [77]. Furthermore, in brown teff, 4-oxo-β-apo-13-carotenone was identified by mass spectrometry (MS), which can act as an antioxidant and modulate antioxidant response genes, indicating its potential role in ameliorating diseases triggered by oxidative stress [90].

### 3.6. Teff—A Good Source of Dietary Fiber

This crop is rich in dietary fiber, comprising both soluble and insoluble components, which are crucial for digestion and metabolism. Soluble fiber, such as beta-glucans, acts as a prebiotic, promoting the growth of beneficial gut bacteria and generating short-chain fatty acids that help maintain a healthy gut barrier [87,91]. Insoluble fiber increases stool bulk, helping regular bowel movements and lowering the risk of colorectal issues and diseases [76]. Additionally, teff is high in fiber and helps to maintain a low glycemic index, making it a good option for managing blood sugar and reducing the risk of type 2 diabetes [38]. These bioactive components make teff a beneficial food that may help prevent chronic illnesses such as heart disease, diabetes, and osteoporosis [92].

## 4. Health Benefits Associated with Teff Consumption

### 4.1. Reduces Cholesterol Levels and Supports Heart Health

Teff is recognized for its high nutritional content, which supports heart health by lowering cholesterol levels. It contains a significant amount of dietary fiber, especially soluble fiber, which can help lower low-density lipoprotein levels. A study by Gebremariam et al. [12] found that the fiber in teff binds to bile acids in the gut, facilitating their removal and promoting the body’s use of cholesterol to produce additional bile acids. This action can lower overall cholesterol levels. Furthermore, teff contains healthy unsaturated lipids, including omega-3 and omega-6 polyunsaturated fatty acids, which can help improve lipid profiles [38].

Research by Baye et al. [75] shows that teff’s blend of fiber, healthy lipids, and minerals makes it an excellent grain for heart health. Teff is rich in magnesium, a mineral vital for heart health. Magnesium helps regulate blood pressure and ensures the heart functions properly, thereby reducing the risk of heart disease. Additionally, teff is abundant in antioxidants, particularly in polyphenols and flavonoids, which aid in reducing oxidative stress and inflammation, key contributors to heart health disease [93].

### 4.2. Stabilizes Blood Sugar Levels and Benefits for Low Glycemic Index

This tropical grain is rich in fiber, particularly resistant starch, which helps to stabilize blood sugar levels and prevents rapid fluctuations in carbohydrate digestion and glucose release into the bloodstream. This quality not only benefits individuals with diabetes but also aids in preventing insulin resistance and metabolic syndrome. A study by Zhu [38] revealed that meals containing teff may help manage diabetes and reduce the risk of type 2 diabetes. The low glycemic index of teff makes it a beneficial grain for individuals with diabetes or anyone aiming to manage their blood sugar levels. Teff’s complex carbohydrates are digested slowly, preventing a rapid increase in blood sugar. Research conducted by Yilmaz [42] disclosed that foods made with teff resulted in lower post-meal blood glucose levels compared to other grains, confirming its role in diabetic diets.

### 4.3. Supports Bone Density and Strength Health

Teff is also rich in calcium, a vital mineral for maintaining bone and tooth strength. This grain has greater bioavailability, exceeding that of several other crops in calcium content, which benefits individuals who may not receive enough calcium. Notably, the calcium levels in teff, as revealed by Baye et al., are as high as those in milk, suggesting that teff may be favorable for bone health, particularly in regions with low dairy utilization [80]. Teff also contains magnesium, phosphorus, and zinc, which are essential nutrients for maintaining bone health. These minerals strengthen and solidify bones, assisting in preventing osteoporosis and fractures. Dijkstra’s research as shown that eating teff on a regular basis can improve bone health. This is especially true for kids, pregnant women, and older people [94].

### 4.4. Immune System Support

Teff has zinc, iron, and other micronutrients that help the body fight off infections and keep the immune system healthy. Zinc is important for making, maturing, and signaling immune cells, and iron is important for their metabolism because it moves oxygen and makes energy [95]. Teff also has antioxidants that lower oxidative stress, which can be bad for the immune system. Jaroszewska et al. [96] showed that teff’s polyphenols and flavonoids help it fight inflammation and boost the immune system, making it a good food for building up resistance to disease. Gebremariam et al. [12] assert that traditional methods, including fermentation, are thought to improve the bioavailability of minerals in teff and help boost immunity.

## 5. Teff Applications in Food Products

The functional characteristics of the teff make it valuable for different traditional and modern food applications [6]. Teff has small starch granules, and an increased amylose content results in slow gelatinization and water absorption, thereby enhancing the texture and shelf life of baked products [38,97]. It lacks gluten, which is beneficial for individuals with celiac disease. However, it must be combined with hydrocolloids or proteins to obtain an elastic dough for gluten-free bread. However, teff binds together better than many other gluten-free grains, making it an excellent option for gluten-free recipes [2]. Moreover, teff contains a significant amount of dietary fiber, which contributes to the dough’s texture and helps retain moisture. Its phenolic compounds also aid in extending the shelf life of products by hindering the oxidation of lipids [44]. Fermenting teff flour (injera) increases nutrient intake and produces organic acids that lower pH, making it safer from microorganisms [2]. However, teff has a tiny particle size and absorbs a notable amount of water, which can complicate large-scale processing. This highlights the need to improve milling and hydrating practices [91].

### 5.1. Traditional Uses

Teff has been a staple food crop in the traditional diets of Ethiopia and Eritrea for centuries. It is primarily used to make injera, a sourdough pancake-like flatbread, a staple of Ethiopian cuisine (Table 5). Soaking teff flour enhances its nutritional value by making the minerals more readily absorbable by the body, allowing it to derive greater benefits from teff. Teff primarily contributes to injera through its starch granules and natural fermentation, which share injera’s distinctive sour flavor and light texture [12,98]. Teff can also be ground into flour to create nutritious porridges, such as Genfo, and fermented drinks, like Tella, as well as non-alcoholic alternatives. As a widely consumed staple food, teff is utilized in various ways to address micronutrient deficiencies due to its high iron and calcium content [35,80].

### 5.2. Modern Uses

Teff is gaining worldwide recognition as a valuable ingredient in innovative food products, and it is widely used as a substitute for wheat flour in baking. The lack of gluten and its good water-binding capacity make it an excellent choice for gluten-free baked goods, such as bread, muffins, and cookies, as it enhances texture and retains moisture better than refined grain alternatives [12,38]. Teff can be used in extruded snacks, breakfast cereals, and energy bars. Its nutty flavor and crunchy texture are highly appreciated when added to various food matrices. This trend of snacks features a low glycemic index and high fiber content, aligning with the trend of snacks [38,99] (Table 6). This crop grain also offers a beneficial combination of amino acids, slow-digesting carbohydrates, and abundant minerals, making it an excellent alternative in sports nutrition products, such as protein bars and recovery drinks. Its antioxidants and prebiotics boost its attractiveness in functional foods designed to promote gut health and metabolic wellness [80].

## 6. Challenges and Future Prospects

Teff is a commonly used food in Ethiopia and Eritrea, and this lack of global awareness hinders its demand in the international market. Gebremariam et al. argues that the potential of teff grains is often underrated due to unmarked products and a lack of knowledge [12]. Focused awareness campaigns, food demonstrations showcasing teff, and collaboration with health organizations can increase global acceptance and demand for teff. Moreover, gluten-intolerant individuals consume these grains safely, and publishing studies that boost the benefits of teff in scientific journals and media could further support consumer demand [38]. Small-scale farmers in Ethiopia primarily produce teff grains, but outdated farming practices and limited access to modern techniques hinder production growth. As Minten [109] noted, teff yields are especially lower than those of other grains because farmers rely on rainfall and use fewer fertilizers or better seeds. To enhance teff production, there is a high need to invest in farming tools, such as irrigation, and develop improved teff varieties through breeding [110].

Since the crop is adapted in more areas with climate and soil environments similar to those in eastern Ethiopia. Pilot initiatives in the U.S., Australia, and Europe show that teff can adapt effectively in these places [111]. Increasing worldwide production could help to meet the rising requirement for wheat alternatives and healthy grains. Nonetheless, this will require solutions to ensure seed accessibility, assist knowledge sharing, and supply market access for farmers [112].

Current studies show that teff grains may offer various health benefits beyond their nutritional value, including antioxidant, anti-inflammatory, and gut-supporting properties. Teff is rich in essential nutrients, including iron, calcium, zinc, and others. However, these minerals may not be easily absorbed due to anti-nutritional compounds, such as phytates. Research by Baye et al. suggests that fermentation, germination, and milling techniques can reduce phytate levels and enhance mineral absorption [80]. Additionally, adding nutrients and mixing flour with teff could increase its nutritional value and assist individuals who lack essential micronutrients [11].

## 7. Conclusions

Teff (*E. tef*) is recognized for its rich nutrient content and numerous health benefits, including high protein levels, fiber, and essential minerals such as iron and calcium. This makes it a superfood that fights malnutrition. With its gluten-free composition, low glycemic index, and antioxidants, teff is suitable for special diets, particularly for individuals with celiac disease, diabetes, or those focused on maintaining their health. Although traditionally used to make injera, teff is now utilized in gluten-free baked goods, snacks, and other functional foods, reflecting the global interest in improved nutrition.

However, teff’s growing popularity is hindered by a lack of knowledge, limited smallholder farming practices, and inadequate processing methods. Improving production through better farming techniques, research, and marketing can help position teff as a vital food source for food security and sustainable diets. Through cooperation with stakeholders, teff can evolve from a local food to a grain that is appreciated worldwide, linking tradition with modern health trends.

## Figures and Tables

**Table 1:** The nutritional profile of teff compared to other common cereals per 100 g.

Component	Teff	Wheat	Rice	Maize	Oat	References
Calories (kcal)	357	375	359	365–385	389–410	[6,34,35,36,37]
Proteins (g)	12.8–20.9	10–13.2	6–8.37	6.4–9	7.7–17	[5,6,35,36,37,38]
Carbohydrates (g)	57.3–86	72–75.9	74.3–85	73–76	66–70	[6,35,36,37,39,40]
Fat (g)	2.4–5.1	1.5–2.5	0.5–1.0	1.4–4.5	6–7.5	[6,35,36,37,40]
Total dietary fibers (g)	3.7–12.2	3.9	0.5–1	3.1–9	4.5–11	[6,35,36,37]
Calcium (mg)	180	25–35	58.1	2–7	50–70	[6,35,36,37,41,42]
Iron (mg)	7.63	3.0–4.5	0.2–0.8	1.5–2.5	4.5–5.5	[6,31,32,33,43,44]
Magnesium (mg)	184	120–140	37.8	35–127	43.9	[6,11,36,37,45]

**Table 2:** The amino acid content in teff compared to other common cereals (g/100 g).

Amino Acid	Tef [57,58]	Wheat [57,59,60,61]	Quinoa [60]	Maize [57,60]	Rice [57,62]
Aspartic Acid	6.4–7.9	4.9	3.7	5.3	9
Threonine	3.6–3.8	2.9	5.7	3.2	3.8
Serine	4.0–4.1	4.9	1.7	4.8	5.1
Glutamic Acid	18.3–21.8	33.8	8.8	18.3	18
Proline	3.7–8.2	10.6	1.8	11.2	NA
Glycine	3.1–6.1	4.5	3.0	2.7	4.6
Alanine	5.9–10.1	3.7	2.2	8.2	5.8
Valine	5.0–5.9	4.5	1.0	5.2	5.2
Methionine	1.8–3.3	1.4	0.3	0.91	2.9
Isoleucine	3.3–3.4	3.5	0.8	2.6	2.6
Leucine	6.1–8.1	7.4	0.8	13.3	8.3
Tyrosine	3–3.2	1.6	1.2	4.6	5.5
Phenylalanine	3.4–5.0	4.9	1.5	4.9	5.7
Histidine	2.8–2.9	2.2	2.2	4.2	2.4
Lysine	3.0–5.6	2.7	2.4	2.1	3.1
Tryptophan	1.3	NA *	NA *	NA *	1.5

* NA—not applicable.

**Table 3:** Fatty acids profile of teff [*Eragrostis teff* (Zucc.) Trotter].

Fatty Acids	Variety of Teff (% of Total Fatty Acid)		Functional Properties	References
	White Teff	Brown Teff		
Linoleic acid	17–39	27–34	body’s growth and development reduce inflammation reduce autoimmune diseases decrease risk of cardiovascular disease, cancer, and type 2 diabetes	El-Alfy et al. [5] Amare et al. [43] Baye et al. [44] Yisak et al. [65]
Oleic acid	20–24	17–24		
α-Linolenic acid	14–21	17–22		
Palmitic acid	9–17	11–14		
Stearic acid	10–13	19–12		

**Table 4:** Comparing the mineral content of teff grain with other common cereals (mg/100 g).

Mineral	White Teff	Red Teff	Mixed Teff	Maize	Sorghum	References
Calcium	150–180	140–170	145–175	2–7	13–28	[6,11,40,44]
Iron	5.2–7.6	7.1–9.3	6.5–8.5	0.5–2.7	3.4–4.8	[6,11,40,44]
Magnesium	160–184	155–180	158–182	35–127	120–165	[6,8,40,44]
Phosphorus	340–429	330–415	335–425	210–330	280–370	[6,11,40,44]
Potassium	390–427	380–415	385–420	270–350	330–363	[6,8,38,44]
Sodium	12–15	10–14	11–14	15–35	6–12	[6,38,40]
Zinc	3.2–4.0	3.0–3.8	3.1–3.9	1.7–2.5	1.6–2.8	[6,11,40,44]

**Table 5:** Comparison of teff, wheat, rice, and oats vitamin content per 100 g.

Vitamin	Teff	Wheat	Rice	Oats	References
Vitamin B1 (mg)	0.39	0.12–0.30	0.02–0.07	0.46–0.76	[6,80,81]
Vitamin B2 (mg)	0.27	0.03–0.12	0.01–0.03	0.11–0.17	[6,38,81,82]
Vitamin B3 (mg)	3.363	5.0–5.6	1.6–2.1	0.9–1.3	[6,80,83]
Vitamin B6 (mg)	0.482	0.30–0.40	0.05–0.16	0.10–0.12	[6,81,82]
Vitamin B9 (µg)	18–36	28–44	2–8	32–56	[80,82]

**Table 6:** Exploration of teff’s versatility in food applications.

Food Application	Description	References
Injera	Ethiopian traditional flatbread with a spongy texture	[100,101]
Porridge	Nutrient-dense porridge often consumed for breakfast	[102,103]
Flour	Teff flour was utilized as a gluten-free flour alternative in different baking recipes	[104]
Pancakes/Waffles	Teff flour was included in pancake or waffle batter for a dietary and gluten-free breakfast	[105]
Bread	Teff flour is used in bread preparation to improve nutritional value and provide an impressive flavor and texture.	[105,106]
Cookies	Teff cookies were dark in color and alternative in gluten-free nature, contributing a nutty flavor and fine texture.	[107]
Cereal bars	Teff grains were combined with nuts, dried fruits, and sweeteners to make gluten-free cereal bars filled with nutrients.	[108]
Sports products	Protein bars, energy gels, and rehabilitation shakes using teff’s slow-release carbohydrates and minerals	[80]
Couscous	Teff grains were cooked and exhibited a nutritious choice compared to traditional wheat couscous.	[104]
Teff based beverages	Teff flour was utilized to make different beverages containing smoothies, teas, and fermented drinks	[109]
